# Paliperidone and aripiprazole differentially affect the strength of calcium-secretion coupling in female pituitary lactotrophs

**DOI:** 10.1038/srep08902

**Published:** 2015-03-10

**Authors:** Marek Kucka, Melanija Tomić, Ivana Bjelobaba, Stanko S. Stojilkovic, Dejan B. Budimirovic

**Affiliations:** 1 Section on Cellular Signaling, National Institutes of Child Health and Human Development, NIH, Bethesda, MD 20892; 2 Clinical Trials Unit, Kennedy Krieger Institute/Johns Hopkins School of Medicine, Baltimore, MD 21205

## Abstract

Hyperprolactinemia is a common adverse *in vivo* effect of antipsychotic medications that are used in the treatment of patients with schizophrenia. Here, we compared the effects of two atypical antipsychotics, paliperidone and aripiprazole, on cAMP/calcium signaling and prolactin release in female rat pituitary lactotrophs *in vitro*. Dopamine inhibited spontaneous cAMP/calcium signaling and prolactin release. In the presence of dopamine, paliperidone rescued cAMP/calcium signaling and prolactin release in a concentration-dependent manner, whereas aripiprazole was only partially effective. In the absence of dopamine, paliperidone stimulated cAMP/calcium signaling and prolactin release, whereas aripiprazole inhibited signaling and secretion more potently but less effectively than dopamine. Forskolin-stimulated cAMP production was facilitated by paliperidone and inhibited by aripiprazole, although the latter was not as effective as dopamine. None of the compounds affected prolactin transcript activity, intracellular prolactin accumulation, or growth hormone secretion. These data indicate that paliperidone has dual hyperprolactinemic actions in lactotrophs i) by preserving the coupling of spontaneous electrical activity and prolactin secretion in the presence of dopamine and ii) by inhibiting intrinsic dopamine receptor activity in the absence of dopamine, leading to enhanced calcium signaling and secretion. In contrast, aripiprazole acts on prolactin secretion by attenuating, but not abolishing, calcium-secretion coupling.

Anterior pituitary lactotrophs secrete high levels of prolactin (PRL) in the absence of any hormone action *in vitro*, and such hyperprolactinemia (HPRL) is driven by spontaneous electrical activity and the accompanying voltage-gated calcium influx (VGCI). *In vivo* HPRL is caused by i) ectopic pituitary grafts, ii) inhibition of dopamine (DA) release from the hypothalamus, iii) the lack of expression of functional DA receptors in lactotrophs, and iv) the pharmacological application of DA antagonists^1^. Under physiological conditions, DA attenuates the strength of calcium–secretion coupling by activating the G_i/o_-coupled D2 receptors expressed in lactotrophs, leading to the inhibition of VGCI and reduced PRL release^2^,^3^,^4^. There are three levels in the control of PRL release by DA: (i) down-regulation of cAMP production, (ii) inhibition of spontaneous electrical activity and accompanied VGCI, which is in part dependent on cAMP levels, and (iii) modulation of the exocytotic pathways downstream of VGCI^5^,^6^,^7^,^8^,^9^. Lactotrophs also express thyrotropin-releasing hormone (TRH) calcium mobilizing receptors, whereas their sister cells somatotrophs do not express TRH and DA receptors^1^.

For decades, HPRL has been recognized as a common side effect of antipsychotic medications used in the treatment of patients with schizophrenia^10^. Moreover, HPRL has clinical consequences on physical health in both short- and long-term treatments (Szarfman et al., 2006; Montejo et al., 2008) in youth (Montejo et al., 2008; Roke et al., 2009) and adults alike (Peuskens, 2014). Among atypical antipsychotics, risperidone (RIS), a D2 receptor higher affinity full-antagonist, poses the greatest risk for marked and sustained HPRL^11^,^12^. In fact, paliperidone (PAL), the 9-hydroxy main metabolite of RIS, is the main causative factor of HPRL^12^,^13^,^14^,^15^,^16^. In contrast, aripiprazole (ARI) is a PRL-sparing drug, labeled as the first D2/D3 receptor partial agonist with a unique pharmacological profile^17^,^18^. Others have suggested that ‘the atypicality' of ARI is most likely combined with its actions on non-DA receptors^19^,^20^,^21^. Furthermore, as the clinical utilization of ARI has increased, a number of clinical studies have found that ARI is a PRL-normalizing agent^22^,^23^,^24^, which has begun to be tested in controlled clinical trials (http://clinicaltrials.gov/show/NCT01338298).

Yet, our understanding of the pathophysiology of antipsychotic-induced HPRL is incomplete. This includes the lack of mechanistic (basic) studies, which could help to distinguish the direct effects of PAL and ARI on human lactotrophs from those that might involve altered hypothalamic secretion of DA and other PRL-inhibiting and/or PRL-stimulating factors, as well as the contribution of receptors other than DA in the action of these drugs. We are also unaware of existing data on the effects of PAL on PRL release in isolated animal pituitary cells.

To address these questions, we studied the direct effects of PAL and ARI on lactotroph function using a primary culture of female rat anterior pituitary cells as a model system. Both static cultures and perifusion experiments were used to study effects of these compounds on cAMP production and PRL secretion. In cells in static cultures, cAMP was measured intracellularly and extracellularly to account for a substantial cAMP release by cAMP transporters expressed in pituitary cells^25^. We compared the concentration-dependent effects of PAL and ARI on spontaneous and DA-regulated PRL and growth hormone (GH) synthesis and release *in vitro*, the latter used as a control for specificity of compound actions. We also examined the status of cAMP/calcium signaling under these experimental conditions.

## Results

### DA and ARI but not PAL inhibits PRL release and cAMP production

**Figure 1:**
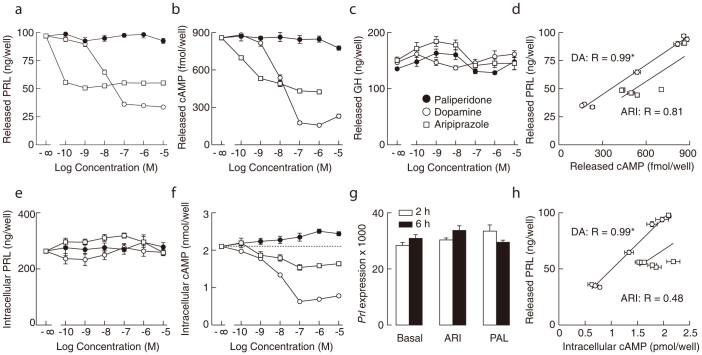
Concentration-dependent effect of dopamine (DA), aripiprazole (ARI), and paliperidone (PAL) on prolactin (PRL), growth hormone (GH), and cyclic AMP (cAMP) release and intracellular content in pituitary cells in static cultures. (a and b) Both ARI and DA inhibited PRL (a) and cAMP (b) release, whereas PAL had no obvious effects on either. Notice that 1 μM ARI was less effective than 1 μM DA in inhibiting PRL and cAMP release. (c) No effect of DA, ARI, or PAL application on GH secretion. (d) A highly significant correlation between cAMP and PRL release in DA-treated cells (open circles) and the lack of significant correlation between these parameters in ARI-treated cells (open squares). (e) No effect of DA, ARI, or PAL on intracellular PRL content. (f) ARI and DA decreased intracellular cAMP content in a concentration dependent manner, whereas PAL increased it at higher concentrations. (g) The lack of a 2 h and 6 h treatment with 1 μM ARI and 1 μM PAL on PRL gene (*Prl*) expression, shown as relative to the expression of the housekeeping gene *Gapdh* (100%). (h) A highly significant correlation between intracellular cAMP content vs. released PRL in DA-treated cells and the lack of correlation between these parameters in ARI-treated cells. Correlation analysis shown in panels (d) and (h) was performed as described in the Methods, and data points are derived from panels (a), (b), and (f). *P < 0.01.

In static anterior pituitary cultures, cells were releasing approximately 100 ng/ml/h hormone (Fig. 1a), confirming that spontaneous calcium secretion coupling was operative. In these cells, we examined the effects of DA, ARI, and PAL in concentrations ranging from 100 pM to 10 μM on hormone secretion and cAMP production during a 60-min incubation.

DA inhibited basal PRL secretion in a concentration-dependent manner with an EC_50_ of ~10 nM. ARI applied at a 0.1 nM concentration also inhibited basal PRL release to approximately 50% of that observed in controls, but further increases in concentration did not produce additional inhibition of hormone release. Under these experimental conditions, no obvious effect of PAL on PRL release was observed (Fig. 1a).

ARI and DA (but not PAL) also inhibited cAMP release from the cells in a concentration-dependent manner; DA was more effective but less potent than ARI (Fig. 1b). Interestingly, ARI had a different potency of inhibiting PRL and cAMP release (Fig. 1a vs. b). In contrast, none of the ligands affected basal GH secretion at the concentrations applied (Fig. 1c), indicating the cell-type specificity of ARI and PAL actions. Moreover, there was a highly significant correlation between decreased cAMP and PRL release when cells were treated with DA (coefficient of correlation R = 0.99; p < 0.01), while for ARI this correlation was weaker (R = 0.81, p > 0.01), reflecting dissociation in the concentration-dependent effects on PRL release and cAMP production.

Neither ligand had an effect on the intracellular PRL content (Fig. 1e), suggesting that PRL synthesis *in vitro* was not affected. Consistently, we did not observe changes in PRL gene (*Prl*) expression in cells treated with PAL and ARI for 2 and 6 h (Fig. 1 g). Several microarray and RNA sequencing analyses of pituitary cells revealed that 2–6 h of treatment is sufficient to observe changes in gene expression in pituitary cells^26^,^27^,^28^,^29^. In contrast, there was a concentration-dependent decrease in intracellular accumulation of cAMP in ARI and DA-treated cells, but DA was a more effective inhibitor of cAMP production. Surprisingly, PAL at higher concentrations stimulated intracellular cAMP accumulation (Fig. 1f), suggesting that D2 receptors exhibit intrinsic activity.

In parallel to the correlation between released cAMP and PRL levels (Fig. 1d), there was a linear relationship between intracellular cAMP levels and PRL release (Fig. 1 h), indicating a similar efficacy of DA to inhibit adenylyl cyclase activity and PRL release. Furthermore, there was no significant correlation in ARI-treated cells, reflecting dissociation in the efficacy of this ligand to inhibit adenylyl cyclase and PRL release (Fig. 1 h).

### PAL and ARI reduced the DA-induced inhibition of PRL and cAMP release

**Figure 2:**
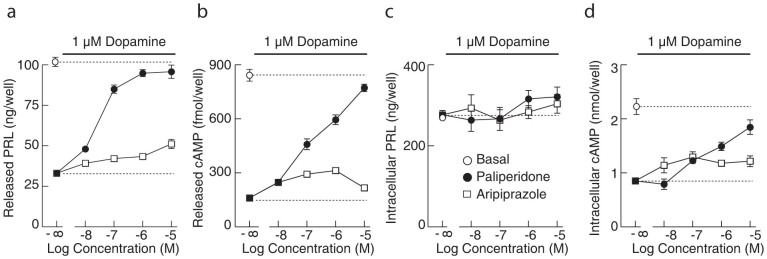
Concentration-dependent effect of ARI and PAL on the released and intracellular content of PRL and cAMP in the presence of 1 μM DA in rat pituitary cells in static cultures. (a and b) ARI partially and PAL completely blocked DA-induced inhibition of PRL (a) and cAMP (b) release. (c and d) Intracellular content of PRL (c) was not affected by DA, ARI or PAL, whereas intracellular accumulation of cAMP (d) was partially rescued by ARI and almost completely by PAL. In this and the following figures, horizontal lines on the top of each panel indicate the duration of treatment. Dotted horizontal lines indicate basal values in the presence (bottom) and absence (top) of DA.

In further experiments with static cultures, we examined the effects of PAL and ARI on DA-induced inhibition of PRL and cAMP release. Figure 2 depicts a concentration-dependent response effect of ARI and PAL in the presence of 1 μM DA on PRL and cAMP release during a 60 min incubation. Increasing the concentration of ARI reduced the inhibitory effect of DA on PRL release, maintaining secretion to a comparable level to that observed in the absence of DA (Fig. 1a vs. 2a). In contrast, PAL at high concentrations completely abolished DA-induced inhibition of PRL secretion (Fig. 2a). Similarly, ARI and PAL reduced DA-induced inhibition of cAMP release in a concentration-dependent manner, and PAL was more effective than ARI (Fig. 2b). Consistent with the results shown in Fig. 1, the intracellular content of PRL was not affected by DA, ARI, or PAL at any concentration (Fig. 2c). As for cAMP cell content, ARI and PAL reduced DA-inhibited cAMP production in a concentration-dependent manner, but with different potency and efficacy (Fig. 2d). Together, these data further support the conclusion that the main action of ARI and PAL on lactotroph function is mediated through D2 receptors.

### PAL stimulates and ARI effectively inhibits PRL release in perifused pituitary cells

**Figure 3:**
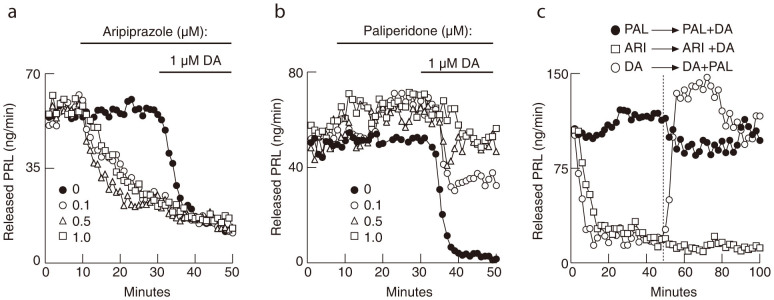
Concentration-dependent response effect of ARI and PAL on PRL release in perifused pituitary cells. (a) ARI inhibited PRL release in all concentrations tested (0.1, 0.5 and 1 μM) and the subsequent application of 1 μM DA further down-regulated hormone release. (b) PAL alone slightly increased PRL release and blocked the inhibitory effect of 1 μM DA on hormone release in a concentration dependent manner; PAL was used at concentrations of 0, 0.1, 0.5, and 1 μM, as indicated. (c) ARI inhibited PRL secretion to comparable levels as DA, whereas DA was unable to inhibit PRL release in the presence of PAL (closed circles); PAL was able to reverse DA inhibitory action with a transient overshot (open circles). All drugs were applied at a 1 μM concentration. The vertical dotted line indicates the moment of switch in drug application as indicated above the line.

In further experiments, we characterized the effects of ARI and PAL, with and without DA, on PRL secretion and cAMP production in perifused pituitary cells. In the absence of DA, a HPRL mode of secretion was observed (50–90 ng/min PRL released) (Fig. 3). In parallel to data in static cultures (Fig. 1a), ARI inhibited PRL release at all concentrations applied (0.1, 0.5, and 1 μM) with a similar rate. The level of inhibition was more profound than that observed in static cultures, and application of DA in the presence of ARI caused only a small additional inhibition (Fig. 3a). In contrast, a small stimulatory effect of PAL on basal PRL release was observed. Furthermore, PAL attenuated the inhibitory effects of DA on PRL release in perifused pituitary cells in a concentration-dependent manner (Fig. 3b), similar to that observed in static cultures (Fig. 2a). This effect suggests that PAL acts as a full D2 receptor antagonist.

Figure 3c depicts the effects of ARI and PAL on PRL release with and without DA. 1 μM ARI (open squares) inhibited PRL secretion in a similar fashion as 1 μM DA (open circles) during the first 45 min application; applying 1 μM DA to cells pre-treated with 1 μM ARI caused only a small additional inhibition of PRL secretion (45–80 min treatment). PAL applied alone at a 1 μM concentration had a delayed stimulatory effect on basal PRL release, as shown in Fig. 3b, which was abolished by application of 1 μM DA (Fig. 3c, closed circles). In contrast, applying 1 μM PAL to cells pre-treated with 1 μM DA blocked the inhibitory effect of DA on PRL release with a transient overshoot (Fig. 3c, open circles).

### DA extends ARI-inhibited cAMP production and PAL facilitates cAMP production

**Figure 4:**
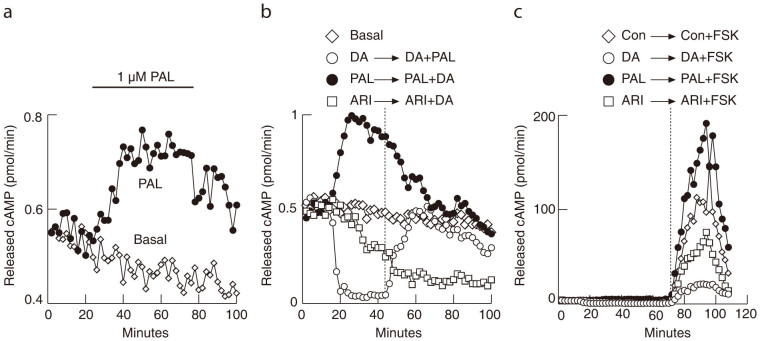
The effect of ARI and PAL on cAMP release in perifused rat pituitary cells. (a) PAL increased cAMP release (20–80 min application) compared to the baseline levels (0–20 min). The washout of PAL was accompanied with a gradual but incomplete return of cAMP release to basal levels during the 20 min washout period. *Basal* indicates cAMP release in untreated cells (open diamonds). (b) ARI (open squares) decreased cAMP release less effectively than DA (open circles). Applying DA to cells pre-treated with ARI further inhibited cAMP production. In contrast, PAL increased cAMP release from cells (closed circles). The addition of DA diminished this increase to basal levels only. Applying PAL to cells pretreated with DA diminished the inhibitory effect of DA on cAMP release (open circles). (c) Application of forskolin, an adenylyl cyclase activator, tremendously facilitated cAMP release (open diamonds). ARI decreased forskolin-stimulated cAMP release (open squares) in a similar fashion but less effectively than DA (open circles), whereas PAL increased forskolin-stimulated cAMP production (closed circles). All drugs were applied at a 1 μM concentration. The vertical dotted line indicates the moment of switch in drug application as indicated above the line.

In perifused pituitary cells, 1 μM PAL increased basal cAMP release. Washout of this ligand caused a gradual return of release towards pre-stimulatory levels (Fig. 4a, closed circles). The stimulatory effect of PAL on cAMP release was also abolished during the co-application with 1 μM DA (Fig. 4b, closed circles), while at the same time PAL completely abolished DA-induced inhibition of cAMP release (Fig. 4b, open circles). PAL also amplified forskolin stimulated cAMP release (Fig. 4c, closed circles). These results further implicated the intrinsic activity of D2 receptors in pituitary lactotrophs, which was silenced by PAL, causing a further increase in cAMP production.

In contrast to PRL release (Fig. 3a), perifusion with 1 μM ARI (Fig. 4b, open squares) was less effective in inhibiting cAMP release than 1 μM DA (open circles), and application of DA in the presence of ARI further inhibited cAMP release. ARI also inhibited forskolin stimulated cAMP release (Fig. 4c, open squares), but less effectively than DA (open circles). These results are consistent with the difference in potency of ARI to inhibit PRL and cAMP release in static cultures shown in Fig. 1a and b, suggesting that not all D2 receptor signaling pathways are activated by ARI with the same potency.

### Modulation of spontaneous VGCI by DA, ARI, and PAL

**Figure 5:**
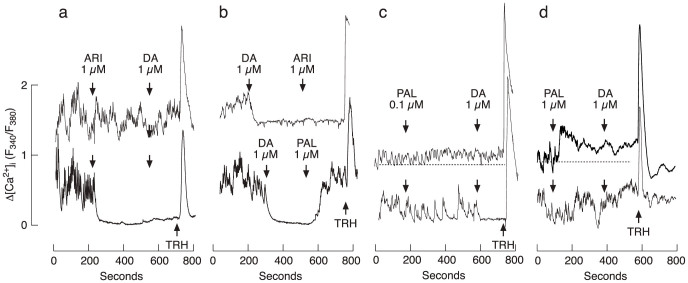
The effect of DA, ARI, and PAL on calcium influx in single rat lactotrophs. (a) Spontaneous calcium transients were blocked by ARI with no further effect of DA in 19 of 31 cells (bottom trace). In the residual cells, ARI did not abolish spontaneous calcium transients but nevertheless blocked DA-induced inhibition of calcium influx (top trace). (b) Once [Ca^2+^]_i_ was inhibited by 1 μM DA, 1 μM ARI could not induce any further effect in 14 of 15 lactotrophs (upper trace). 1 μM PAL recovered spontaneous calcium transients previously blocked by DA in 9 of 10 cells (bottom trace). (c) When applied in 0.1 μM concentration, PAL gradually increased [Ca^2+^]_i_ in a fraction of cells (12 of 48; top trace) and was ineffective in residual cells. In a fraction of non-responders (7 of 48), the subsequent application of 1 μM DA inhibited calcium transients (bottom trace). (d) When applied in 1 μM concentration, PAL rapidly increased [Ca^2+^]_i_ in a fraction of cells (13 of 45; top trace) and was ineffective in residual cells (bottom trace). In all of these 45 cells, PAL blocked further effect of 1 μM DA. Arrows indicate the moment of drug application, and the subsequent compound was added without dilution of the first compound. Horizontal dotted lines in panels c and d indicate baseline [Ca^2+^]_i_.

Figure 5 depicts [Ca^2+^]_i_ measurements in single rat pituitary cells. In lactotrophs, TRH induced a rapid spike response, reflecting calcium mobilization from the endoplasmic reticulum (Fig. 5a–d), whereas DA inhibited spontaneous fluctuations in [Ca^2+^]_i_ (Fig. 5 b), reflecting inhibition of spontaneous electrical activity and accompanied VGCI. In contrast to DA, 1 μM ARI mimicked the action of DA on inhibition of calcium transients in only about 60% of lactotrophs (19 of 31) (Fig. 5a, bottom trace). In the residual cells, ARI was ineffective (Fig. 5a, top trace). However, in both cases DA had no effect on [Ca^2+^]_i_ in the presence of ARI (31 of 31 cells). In DA-treated cells, ARI was also unable to change [Ca^2+^]_i_ in 14 of 15 cells (Fig. 5b, top trace). These results are consistent with the action of ARI on D2 receptors as a partial agonist.

On the other hand, 1 μM PAL reversed DA-induced inhibition of VGCI in 9 of 10 cells (Fig. 5b, lower trace), confirming that it acts as D2 receptor antagonist. In addition, PAL exhibited stimulatory effect on VGCI, but only in a fraction of lactotrophs. When applied in 0.1 μM concentration, PAL slightly and gradually increased [Ca^2+^]_i_ in 12 of 48 TRH-responsive cells (Fig. 5c, top trace). In the residual cells PAL was ineffective and in some of these cells (7 of 48) 1 μM DA inhibited spontaneous calcium oscillations (Fig. 5c, bottom trace). When applied in 1 μM concentration, PAL stimulated rise in [Ca^2+^]_i_ in 13 of 45 (Fig. 5d, top trace) and was ineffective in the residual cells (Fig. 5d, bottom trace). However, 1 μM DA was not able to inhibit calcium fluctuations in any of the lactotrophs treated with 1 μM PAL (illustrated in Fig. 5d). These results are in agreement with data shown in Fig. 4, implicating the PAL-sensitive intrinsic activity of D2 receptors only in a fraction of lactotrophs.

## Discussion

We have demonstrated that VGCI and adenylyl cyclase are spontaneously active and that calcium–secretion coupling is preserved in static cultures and in perifused pituitary cells from female rats in the absence of DA, ARI and PAL. In addition, we have shown that lactotrophs are in a HPRL mode of secretion, a finding that is consistent with previous work by our group^1^,^30^. We also show that two antipsychotics directly modulate cAMP/calcium signaling and PRL release, but not GH release, in the presence and absence of dopamine; PAL amplifies the HPRL mode of lactotroph secretion, and ARI attenuates but does not completely block VGCI-secretion coupling.

The key question is which receptors account for the action of these three ligands? In pituitary lactotrophs, DA acts exclusively through its D2 receptors^6^. In addition to DA receptors, however, PAL also acts as a potent antagonist at 5-HT_2A_, α1 and α2 adrenergic receptors, and H_1_ histaminergic receptors^31^. ARI is not only deemed a partial^17^,^18^ or selective^21^ agonist of D2/D3 receptors, but it also acts as a potent partial agonist at the 5-HT_1A_ receptor^32^,^33^ and 5-HT_2C_ receptor^19^, as well as an antagonist at the 5-HT_2A_^20^,^33^ and 5-HT_7_^34^ receptors.

Our recent RNA-Seq analysis of cultured pituitary cells revealed that they express D2 receptors robustly, whereas the expression of 5-HT_1A_, 5-HT_2A_, 5-HT_2C_, α1 and α2 adrenergic, and H_1_ histaminergic receptors were negligibly expressed or undetectable; the data from RNA sequencing has been deposited with the NCBI Sequencing Read Archive (http://www.ncbi.nlm.nih.gov/sra) and is available under accession number SRA062949. Furthermore, in our experimental conditions serotonin and norepinephrine did not affect basal PRL release (data not shown). This finding indicates that the observed effects of atypical antipsychotics ARI and PAL in cultured pituitary cells reflect their primary actions on D2 receptors. Experiments with co-applications of both ligands also support this conclusion.

Another key question is what is the mode of action of PAL and ARI on D2 receptor? Our data clearly indicate that PAL acts as a full antagonist of D2 receptors. It attenuates or blocks the inhibitory effect of DA on cAMP/calcium signaling and PRL release depending on concentrations of two drugs. We also observed that PAL stimulates basal cAMP production and PRL release in the perifused pituitary cells. In the absence of other PAL-activated receptors in lactotrophs, these data suggest that D2 receptors exhibit intrinsic activity, which contributes to the control of signaling and secretion. Occupancy of D2 receptors by PAL abolished such activity, leading to further facilitation of VGCI, cAMP production, and PRL release. Single cell calcium analysis indicates that this effect was not observed in all lactotrophs, but only in about 30% of these cells, which could reflect the level of D2 receptor expression in individual cells. In contrast, PAL reversed the DA-induced inhibition of calcium transients in practically all lactotrophs.

We also progressed in understanding the nature of ARI action in lactotrophs. ARI is labeled as the D2/D3 receptor partial agonist^17^,^18^. Partial agonists of D2 receptors differ greatly in their ability to induce PRL release, and it has been proposed that this is due to their kinetic properties e.g., dissociation rate and affinity^35^. Our understanding of ligand action as a partial agonist implies that it is less potent and less effective than the native agonist. However, in our experiments, ARI exhibits a significant left shift in concentration dependence of PRL release when compared to DA, although it is less effective in terms of the maximum inhibition of PRL secretion. Additionally, the efficacy of ARI increased in experiments with perifused pituitary cells, which is a more physiological setting to study the effects of drugs on secretion. In contrast, ARI is less potent in inhibiting cAMP production and release when compared to inhibition of PRL secretion, whereas DA inhibits both PRL release and cAMP production/release with similar potency. Finally, ARI blocked DA action in all lactotrophs but mimicked the inhibitory action of DA only in about 60% of cells. We interpret these differences in the action of ARI vs. DA on cAMP signaling and PRL release in support of a previously proposed hypothesis that ARI acts as a selective agonist for D2 receptors^21^.

What is the clinical implication of our *in vitro* study? In humans, both injectable and oral formulations of PAL cause HPRL over short and longer-term treatment periods^36^,^37^,^38^. For example, serum PRL levels were increased in approximately 40% (out of 2831 male and female patients) of subjects treated with PAL. However, in that study there was no significant correlation between monthly dose and proportion of subjects with elevated prolactin levels^16^. In general, 2, 6, 10, and 16 mg of RIS and PAL per day, corresponds to 14, 45, 73, and 110 ng/ml in blood concentration, respectively (Quest Diagnostics Valencia, Nichols Institute, Valencia, CA official laboratory reports issued to clinicians. Director: M. Dugan, MD, FCAP). When treated with 4–6 mg RIS, the steady-state levels of blood RIS concentrations were established within seven days and were accompanied with elevation in serum PRL levels^39^. Treatment with 2–8 mg/day with RIS also elevated serum PRL levels in patients with schizophrenia^40^. In our experiments, the corresponding dose of about 100 nM almost reached the steady-state elevation in PRL secretion in the presence of DA. The results, for the first time, also indicate that the D2 receptor exhibits a small intrinsic activity, which contributes to the control of signaling and secretion (i.e., PAL abolished such activity, leading to further facilitation of PRL release). From the clinical point of view, this is an additional unwanted feature of the compound because it could further worsen HPRL.

When patients were treated with 5–30 mg ARI, the blood concentrations of this compound were in the ranged of 100–400 ng/ml and dehydroarpiprazole in 20–200 ng/ml^41^. The postmortem femoral blood concentrations of ARI in treated patients range from 49 to 690 μg/kg^42^. In that concentration range, in the rat model we observed some stimulatory effects of ARI in DA-treated cells, representing 45–50% of that observed in cells treated with PAL. In further accordance with this, serum PRL levels in patients treated with ARI were decreased or unchanged^43^,^44^,^45^,^46^. Moreover, in RIS-treated patients, ARI also normalized serum PRL levels in a fraction of patients^47^,^48^,^49^.

It is generally accepted that the lack of elevation in serum PRL levels in patients treated with ARI can be accounted for by its actions as a partial D2 receptor agonist. In that respect, ARI may be the drug of choice in patients with antipsychotic-induced HPRL^50^. The observed bidirectional effects of ARI on PRL release, that is, stimulatory in the presence of DA and inhibitory in the absence of DA, are in general agreement with data obtained by others^51^; in immortalized GH_4_C_1_ pituitary cells transiently expressing the short and long form D2 receptors, ARI inhibited forskolin-induced PRL release and cAMP production, albeit less effectively than DA.

The ability of ARI to suppress cAMP production that we report here could be another reason for favoring it over PAL in patients with schizophrenia. Namely, cAMP induces nerve growth factor-inducible gene expression^52^, which is tightly related to the dopaminergic system in the striatum^53^ and has a critical role in antipsychotic induced extrapyramidal side effects in mice^54^. Indeed, other than being less prone to induce HPRL, a recent study revealed that ARI does not cause extrapyramidal side effects, unlike PAL, which is not well tolerated in that respect^12^.

The last key question is there sex specificity in the PAL and ARI action? Several reports indicate that the stimulatory effect of PAL on serum PRL is greater in female than male patients^36^,^55^,^56^,^57^,^58^. Others reported comparable effects in terms of the number of patients responding to treatment with HPRL^16^. An earlier experiment with anterior pituitary cells from male rats cultured in the presence of 10 μM DA showed stimulatory effect of RIS on PRL release in a concentration range of 0.1 to 1 μM^59^. Our experiments with PAL-treated female rat pituitary cells are in agreement with such dose-response, but the amplitude of response in females was about 3-fold higher, a finding consistent with higher PRL secretion in postpubertal female than male rats^30^. In contrast, the sex-specificity of the action of ARI was not studied in the rat model. As for sex specificity of ARI action on PRL release in female patients, a recent clinical study by Veselinovic and colleagues^58^ found significantly higher PRL levels in females than males that received up to 15 mg ARI. In their haloperidol group (up to 3 mg per day), the sex difference reached much higher statistical significance. Together, this clearly indicates the need for further comparative studies on effects of these compounds in females and males.

In conclusion, these data indicate a dual advantage of ARI over PAL in control of lactotroph function. First, we show for the first time that dopaminergic receptors in lactotrophs exhibit small intrinsic (in the absence of ligand occupancy) activity and that binding of PAL silences such activity, leading to enhanced coupling of electrical activity and prolactin secretion. More importantly, PAL effectively blocks DA action in lactotrophs, preserving the HPRL mode of secretion. In contrast, ARI normalizes the secretory output of lactotrophs independently of DA levels by clamping the calcium–secretion coupling between the HPRL and silenced modes.

## Methods

### Chemicals

Fura 2-AM, medium-199, GH Rat ELISA Kit and horse serum were purchased from Life Technologies, Inc. (Grand Island, NY, USA). The primary antibody and standard for the PRL assay were purchased from the National Pituitary Agency and Dr. A. F. Parlow (Harbor-UCLA Medical Center, Torrance, California). cAMP was determined using our specific antiserum. ^125^I-Prolactin and ^125^I-cAMP were purchased from Perkin Elmer Life Sciences (Boston, MA). Unless stated otherwise, all other chemicals were obtained from Sigma (St. Louis, MO, USA).

### Primary culture of anterior pituitary cells

Experiments were performed on cultured anterior pituitary cells from normal post-pubertal female Sprague-Dawley rats obtained from Taconic Farms (Germantown, NY). Euthanasia was performed by asphyxiation with CO_2_, and the anterior pituitary glands were removed after decapitation. Experiments were approved by the NICHD Animal Care and Use Committee. Pituitary tissue was cut into 1 × 1 × 1 mm pieces and treated with trypsin (40 mg/ml) for 15 minutes at 37°C followed by mechanical dispersion. Dispersed pituitary cells were then cultured as mixed cells in medium-199 containing Earle's salts and supplemented with 10% horse serum, penicillin (100 U/ml), and streptomycin (100 μg/ml) (Life Technologies, Inc.). Two types of experiments were performed.

In the static culture experiment, cells were seeded onto poly-D-lysine coated 24-well plates at 0.5 million or 0.25 million cells/well for cAMP and PRL/GH measurements, respectively. Twenty-four hours after seeding, fresh medium supplemented with drug treatment was applied for 1 h; afterwards, medium was removed to analyze changes in cAMP and hormone secretion. To analyze changes in the intracellular content of PRL or cAMP, 0.5 ml of ice-cold 20 mM sodium carbonate buffer (PRL samples) or 100% ethanol (cAMP samples) was added into wells, and plates were frozen at −80°C. Next, plates were scraped using a 1 ml pipette tip and contents transferred into individual tubes. The process of scraping was repeated with 0.5 ml of fresh buffer or ethanol and added to the tube containing the first extract. Cell extracts were centrifuged at 3000 rpm to remove cell debris. The ethanol in the cAMP samples was evaporated and samples were re-suspended in 0.5 ml of PBS containing 0.1% BSA, 1 mM 3-isobutyl-1-1-methylxanthine (IBMX) and acetylated. Samples were stored at −20°C until analysis.

In the perifusion experiment, 12 million cells were cultured with pre-swollen Cytodex-1 beads (Sigma) for 24 hours. On the following day, beads with attached pituitary cells were transferred to 37°C-heated chambers and continuously perifused with warm HEPES-containing medium-199 supplemented with 0.1% BSA and penicillin (100 U/ml) and streptomycin (100 μg/ml). The chambers were perifused for 2 hours at a flow rate of 0.5 ml/min to establish stable secretion. PAL, ARI, DA or combination treatments in the absence or presence of forskolin, an allosteric activator of adenylyl cyclase, were continuously applied to cells and 1-min fractions of perifused medium were collected and stored at −20°C until analysis.

In all experiments for cAMP measurements, the medium was supplemented with 1 mM IBMX, a non-specific inhibitor of cAMP and cGMP phosphodiesterases. Collected samples were immediately acetylated with a 2:1 mixture of triethylamine and acetic anhydride (10 μl/0.5 ml medium) and stored at −20°C until analysis.

### Evaluation of calcium signaling in lactotrophs

Measurements of intracellular calcium concentration ([Ca^2+^]_i_) in single pituitary cells were performed as previously described^5^,^6^. Briefly, the cells of the primary culture of the anterior pituitary cells were plated on poly-L-lysine coated coverslips and bathed in Krebs-Ringer-like medium containing 2.5 μM Fura 2 AM for 1 h at room temperature. After that, the coverslips were washed in Krebs-Ringer-like medium and mounted on the stage of an inverted Observer-D1 microscope (Carl Zeiss, Oberkochen, Germany) with an attached ORCA-ER camera (Hamamatsu Photonics, Hamamatsu City, Japan) and a Lambda DG-4 wavelength switcher (Sutter, Novato, CA). Hardware control and image analysis was performed using Metafluor software (Molecular Devices, Downingtown, PA). Experiments were performed under a 40× oil-immersion objective during exposure to alternating 340 and 380 nm excitation beams, and the intensity of light emission at 520 nm was followed simultaneously in approximately 20 single cells. Changes in [Ca^2+^]_i_ are presented by the ratio of fluorescence intensities F_340_/F_380_. In mixed population of rat female pituitary cells, lactotrophs were identified by their responses to both TRH and dopamine, in contrast to thyrotrophs responding only to TRH^60^.

### Quantitative RT-PCR analysis

Total RNA from pituitary glands or primary pituitary cells was extracted using the RNeasy Plus Mini Kit (QIAGEN). The extracted RNA showed integrity of 28S and 18S rRNA bands by agarose gel electrophoresis and had a 260 nm to 280 nm ratio above 1.85; 1 μg of RNA was reverse transcribed with the Transcriptor First Strand cDNA Synthesis Kit (Roche). Quantitative RT-PCR was performed using LightCycler TaqMan master mix and the LightCycler 2.0 real-time PCR system (Roche Applied Science) and Applied Biosystems predesigned TaqMan gene expression assays for rat glyceraldehyde-3-phosphate dehydrogenase (*Gapdh*; Rn01462662_g1) and PRL (*Prl*; Rn00561791_m1). The target gene expression levels were determined by the comparative 2ˆ[-δδ-cycle threshold] quantification method using *Gapdh* as the reference gene.

### Data analysis and statistics

The results shown represent mean ± SEM values from sextuplicate incubations in one of three independent experiments (static cultures; Figs. 1 and 2), or representative traces from three similar experiments (perifusion experiments; Figs. 3 and 4). For calcium recording, the number of experiments is indicated in the Results section. Linear correlations were calculated using the KaleidaGraph Program (Synergy Software, Reading, Pennsylvania).
